# General practitioners’ management of patients with psychological stress: audit results from Denmark

**DOI:** 10.1186/s12875-020-01137-6

**Published:** 2020-04-20

**Authors:** Jesper Lykkegaard, Anders Prior, Marianne Rosendal

**Affiliations:** 1 Research unit of general practice, Institute of public health, University of Southern Denmark, JB Winsløws vej 9A, DK-5000 Odense, Denmark; 2 Research Unit for General Practice, Bartholins Allé 2, DK-8000 Aarhus, Denmark; 3 Functional Disorders, Norrebrogade 44, DK-8000 Aarhus C, Denmark

**Keywords:** Stress, psychological, Primary health care, General practice, Psychiatry, Psychotherapy, Sick leave

## Abstract

**Background:**

In western countries, psychological stress is among the most common causes of long-lasting sick leave and a frequent reason to consult the general practitioner (GP). This study aimed to investigate how GPs manage patients with psychological stress and how the management is associated with the patient’s sex, the GP’s assessment of causality, and coexisting mental disorders.

**Methods:**

We conducted an audit of consecutive cases in Danish general practice. The GPs used electronic medical records to fill in a registration form for each 18–65-year-old patient with whom they had had at least one consultation regarding stress during the past 6 months. Only patients initially in the workforce were included. Age- and sex-adjusted binary regression was applied.

**Results:**

Fifty-six GPs (61% women) identified 785 cases. The patients’ mean age was 44 years and 70% were women. The cause of stress was considered at least partially work-related in 69% of the cases and multifactorial in a third of cases. The management included sick leave (54%), counselling (47%), pharmaceutical treatment (37%), and referral to psychologist (38%). Compared to women, stress in men was less often considered work-related (RR: 0.84, CI95%: 0.77–0.92) and men were less often sick-listed (RR: 0.83 CI95%: 0.73–0.96) but were more often prescribed tranquilizers (RR: 1.72 CI95%: 1.08–2.74).

**Conclusions:**

GPs’ management of patients with stress usually involve elements of counselling, sick leave, referral to psychologist, and medication. Women and men with stress are perceived of and managed differently.

How this fits in

- Psychological stress poses a large burden on western societies, primarily due to sick leave. It is a common reason for consulting the GP, but the provided care has been sparsely described. According to this audit, GPs consider stress in patients in the workforce to be at least partially work-related in 69% of cases and multifactorial in a third of cases. Sick leave, counselling, referral to psychologist, and medication are frequent management strategies often applied together, but there is a gender difference, as GPs less often consider stress in men to be work-related, less often give the men a sick note, and more often prescribe the men tranquilizers compared to women.

## Background

Psychological stress, hereafter referred to as ‘stress’, poses a large burden on societies; stress is often considered work-related, and sick leave is the major cost of the condition [1]. In countries with a large service sector, such as the UK and Denmark, it is the condition causing the highest total number of days on sick leave [1–6]. Furthermore, stress has been associated with adverse outcomes such as development of chronic diseases [7–9] and increased mortality [10].

Patients with stress are frequent in general practice [11–14] and may need to consult the general practitioner (GP) for several reasons. Some may seek evaluation and treatment of psychological and physical symptoms in relation to stress [15, 16]. Others may request a sick note [6]. When GPs act as gatekeepers, the patients may need a referral e.g. to a psychologist, psychiatrist, or specialist in occupational medicine. Nevertheless, there is little knowledge and guidance on effective treatment and the GPs’ management strategies are generally unknown.

Various factors may influence the GP’s management strategy. For example, attitudes towards sick leave may be gender specific [17] suggesting that the patient’s and GP’s sex may be influential. Also, the GP’s assessment of causality and any coexisting mental disorders may influence the management strategy [18].

This study aimed to investigate how GPs manage patients with stress and how the management is associated with the patient’s sex, the GP’s assessment of causality, and coexisting mental disorders.

## Material and methods

### Design

All GPs in the Region of Southern Denmark were invited to a one-day seminar on stress. As a mandatory preparation, the GPs reported characteristics of all their stress cases during the past 6 months. Participating GPs were paid for 2 hours of work on data collection and partially reimbursed for absence from their clinic during the seminar.

### Setting

Denmark has 5.7 million citizens. The healthcare system is publicly paid and includes free access to GP services. About 98% of the population is listed with a general practice. The GPs receive capitation fees and fees for services, including a fee equal to about three times the normal consultation fee for counselling for psychological conditions. No specific type of therapy is required. Patients with stress can have psychotherapy in three other ways. First, the patient can pay for consulting a psychologist. With a GP referral, the public healthcare system remunerates about half of the expenses for psychologist treatment. However, stress is not on the list of approved conditions for issuing a referral. These include depression, anxiety, or experiencing a specified significant psychological trauma. Many Danes hold a health insurance that pays for psychologist treatment of stress, most of them requiring the GP’s written advice to consult a psychologist, but not necessarily in the form of a referral. In this paper “referral to psychologist” includes the GP’s mere advise to consult a psychologist. Second, the municipality provides a stress program for patients at high risk of unemployment, not requiring a GP referral. Third, the hospital departments of occupational medicine provide psychologist treatment requiring a GP referral. The first year on sick leave is compensated by the local municipality and the employer. All GPs use electronic medical records (EMR). The majority of GPs code consultations using the International Classification of Primary Care second edition (ICPC-2-R) [19].

### Identification of patients

Participating GPs were mailed an EMR search procedure to create a list of patients for the audit. The procedure identified all patients aged 18–65 years with a consultation during the period from 1 October 2015 to 31 March 2016 coded with one or more symptoms or diagnoses indicating stress. The included age group was chosen based on the age of majority and retirement in Denmark. The searched ICPC-2-R codes included among others anxiety, depression, sleeping problem, work problem, and non-specified psychological problem (see supplementary). The GPs were asked to review the list and select the patients whom they considered to have stress based on information in the EMR and any further information the GP had. For each selected patient, they filled in a registration form on paper (see supplementary). Patients were excluded if not in the workforce at the beginning of their stress symptoms.

### Data

The GPs recorded the patients’ age, sex, the GP assessed causes of stress, co-existing psychiatric diagnoses, and whether the management had included respectively counselling, sick leave, referral to psychologist, enrolment in the municipality stress program, referral to a hospital department of occupational medicine, and report of suspected work-related disease to the Occupational Health- and Safety-Administration. Furthermore, the GPs looked in the patient’s prescription records which are real-time updated containing all prescriptions made to the patient by any physician in the Danish healthcare system. The GPs recorded whether the patient had prescribed antidepressants, tranquilizers (benzodiazepines and z-drugs e.g. zopiclone or zolpidem), and antipsychotics, respectively.

### Analyses and statistics

The patients were divided into a young group (18–34 years), a middle-aged group (35–54 years), and an older group (55–65 years). The proportions of patients having had each element of management were compared in relation to age, sex, the considered cause of stress, and co-existing psychiatric diagnoses. Binary regressions adjusted for age group and sex were used to test for statistically significant differences in the application of each management element. Risk ratios (RR) were calculated with 95% robust confidence intervals (CI95%) considering the patients’ clustering in GP practices. Significant (*p* < 0.05) associations were further analysed, respectively, stratifying the patients by the GP’s sex and age dichotomized at the median (50 years). All analyses were performed in STATA Release 15 (STATACorp, College Station, TX, USA).

## Results

Invitations were mailed to all GPs in the region of Southern Denmark (*N* = 807), and 59 GPs (7.3%) from 34 practices (9.3%) participated in the audit. The participants’ average age, the number of listed patients, and the proportion of solo practitioners did not differ significantly from that of the total region, but more female GPs participated (61% vs 49%) [11]. One practice with three GPs was excluded for reporting twice the proportion of patients with stress compared to the practice with the second highest proportion. The remaining 33 practices (56 GPs) recorded 1066 patients with stress. Of these, 53 were excluded due to missing data and 228 were excluded for not being in the workforce, leaving 785 patients for the analyses.

**Fig. 1:**
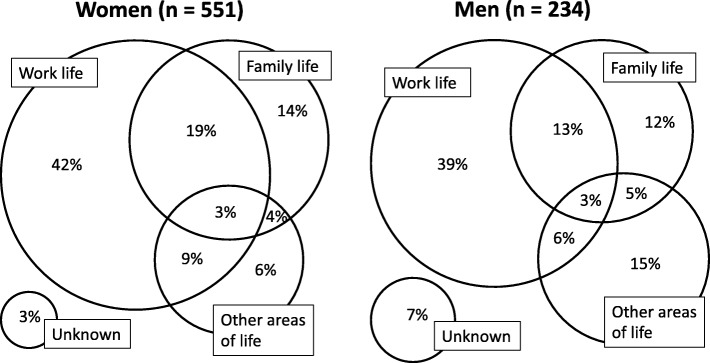
The general practitioner’s assessed cause of psychological stress in 785 patients in the workforce. ‘Other areas’ includes among others stress from having a physical disease

The majority of patients were women (70%) and 35–54 years old (59%). Overall, work (69%) and family (39%) were the areas of life that the GP most often assessed to harbour a cause of the stress (Table 1). The stress was considered multifactorial in about one third of the cases (Fig. 1). Stress was less often considered work-related in men than in women (RR 0.84, CI95% 0.77–0.92), and less often family-related in the 55–65-year-olds compared to the 18–34-year-olds (RR 0.72, CI95% 0.55–0.94).

**Table 1:** The general practitioner’s causality assessment and management of psychological stress according to the patient’s sex and age

Age and sex of the patients		Total	Female (ref)	Male	18–34 yrs. (ref)	35–54 yrs	55–65 yrs
Number of patients		785	551	234	167	464	154
GP assessed causes of stress	Work	69%	74%	61%^*^	63%	71%	69%
	Family	39%	41%	33%	41%	41%	29%^*^
	Physical disease	11%	10%	13%	10%	11%	13%
	Other cause	12%	11%	15%^*^	20%	9%^*^	12%
	Unknown	4%	3%	7%^*^	5%	3%	7%
Medication	Antidepressants	30%	29%	35%	22%	32%^*^	34%^*^
	Benzodiazep or Z-drugs	8%	7%	12%^*^	6%	8%	12%
	Antipsychotics	3%	3%	4%	3%	3%	5%
	None of the above	63%	66%	58%	72%	62%	56%^*^
Period of sick-leave due to the stress		54%	57%	48%^*^	44%	58%^*^	54%
Counselling in general practice		49%	50%	47%	46%	52%	43%
Referred to psychologist		39%	41%	35%	41%	38%	39%
Referred to dpt. of occupational medicine		6%	7%	5%	3%	7%	7%
Reported to OSHA		7%	7%	5%	4%	8%	6%
Participated in municipality stress program		8%	9%	3%^*^	5%	9%	5%

* p < 0.05 compared to the reference (ref). OHSA, Occupational Safety- and Health-Administration

**Fig. 2:**
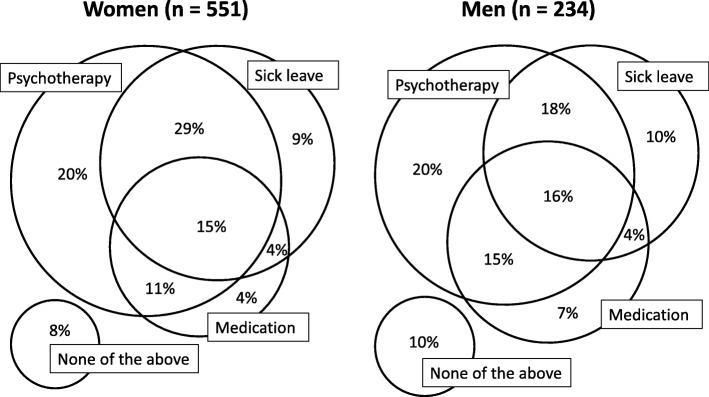
General practitioners’ management of psychological stress in 785 patients in the workforce. Psychotherapy includes counselling, referral to psychologist, enrolment in the municipality stress program, and treatment at a hospital department of occupational medicine

The GPs’ most frequent stress management strategies were sick leave (54%), counselling (49%), referral to psychologist (39%), and medication (37%). In 59% of the cases, the management strategy involved at least two of these, and in only 9% of the cases, none of the four were applied (Fig. 2). The most frequent medication was antidepressants (30%). In men, the management of stress was less likely to include sick leave than in women (RR 0.83, CI95% 0.73–0.96), and men were more likely than women to have tranquilizers prescribed (RR 1.72, CI95% 1.08–2.74).

Certain GP assessed causes of stress were associated with specific elements of management (Table 2). The patients were less often referred to psychologist if the stress was considered work-related (RR 0.83, CI95% 0.70–0.97) and more often if it was considered family-related (RR 1.26, CI95% 1.07–1.49). Medication was more likely when the stress was considered related to physical disease (RR 1.76, CI95% 1.47–2.09). More patients were sick-listed if the stress was considered work-related (OR 1.72, CI95% 1.37–2.14) and fewer if family-related (RR 0.68, CI95% 0.58–0.80) or unknown (RR 0.59, CI95% 0.38–0.90). The patients were more often enrolled in the municipality stress program if the stress was work-related (RR 2.55, CI95% 1.23–5.30).

**Table 2:** The general practitioner’s causality assessment and management of psychological stress

GP assessed causes of stress		Work	Family	Disease	Other	Unknown
Number of patients (groups are not exclusive)		542	303	86	94	34
The patients’ mean age in years		45	43	45	41	45
Patients with female sex		74%^↑^	74%	64%	63%^↓^	48%^↓^
Medication	Antidepressants	29%	32%	50%^↑^	43%^↑^	36%
	Benzodiazepines or Z-drugs	7%	7%	12%	13%	12%
	Antipsychotics	3%	2%	10%^↑^	4%	3%
	None of the above	66%	62%	40%^↓^	50%	49%
Period of sick-leave due to the stress		62%^↑^	43%^↓^	52%	43%	30%^↓^
Counselling in general practice		51%	52%	53%	53%	45%
Referred to psychologist		37%^↓^	45%^↑^	42%	35%	36%
Referred to dpt. of occupational medicine		9%^↑^	3%^↓^	3%^↓^	2%^↓^	1%^↓^
Reported to OSHA		9%^↑^	5%^↓^	3%^↓^	3%^↓^	0%^↓^
Participated in municipality stress program		9%^↑^	8%	8%	7%	3%^↓^

*The arrows indicate where the proportions were significantly (age- and sex-adjusted p < 0.05) higher*^*↑*^*or lower*^*↓*^*if the cause of stress was assessed to be present compared to if not. OSHA, Occupational Safety- and Health-Administration*

Only 9% of the cases assessed as work-related were referred to a department of occupational medicine, and 9% were reported to the Occupational Safety- and Health- Administration.

About half of the patients (48%) had a co-existing psychiatric diagnosis, and 10% had more than one. These patients had almost the same age- and sex-distribution and reported causes of stress as the patients without a co-existing psychiatric diagnosis, but management differed with a higher proportion of patients treated with antidepressants and antipsychotic drugs. Patients with more than one co-existing psychiatric diagnosis were more often sick-listed (RR 1.35, CI95% 1.14–1.59). Patients with anxiety more often received counselling with the GP (RR 1.34, CI95% 1.03–1.75), while patients with depression more often were referred to psychologist (RR 1.53, CI95% 1.23–1.90) and enrolled in the municipality stress program (RR 2.96, CI95% 1.48–5.93) respectively compared to patients not having the condition (Table 3).

**Table 3:** General practitioners’ management of stress according to co-existing psychiatric diagnoses

Psychiatric diagnosis coexisting with stress		None (ref)	Anxiety only	Depression only	Other diag only	> 1 diagnosis
Number of patients		376	93	176	60	80
Mean age in years		43	44	44	44	44
Females		72%	69%	67%	72%	69%
GP assessed cause of stress	Work	73%	63%	64%	63%	75%
	Family	37%	42%	43%	35%	34%
	Physical disease	5%	18%^*^	12%^*^	17%^*^	20%^*^
	Other cause	8%	15%	13%	23%^*^	16%
	Unknown	3%	5%	6%^*^	7%	3%
Medication	Antidepressants	4%	43%^*^	73%^*^	18%^*^	54%^*^
	Benzodiazep or Z-drugs	7%	10%	7%	3%	16%^*^
	Antipsychotics	1%	1%	4%^*^	5%^*^	14%^*^
	None of the above	88%	52%^*^	25%^*^	77%^*^	34%^*^
Period of sick leave due to the stress		53%	45%	54%	52%	71%^*^
Counselling in general practice		43%	58%^*^	53%	52%	55%
Referred to psychologist		32%	45%	49%^*^	37%	45%^*^
Referred to dpt. of occupational medicine		7%	5%	5%	3%	9%
Reported to OSHA		6%	2%	7%	8%	14%
Participated in municipality stress program		5%	6%	14%^*^	5%	1%

* p < 0.05 adjusted for age group and sex compared to (ref). OSHA, Occupational Safety and Health Administration

All the above reported associations had similar directions and strengths when retested in the GP sex- and age-specific strata, except that only when the GP was a woman male patients were less likely to go on sick leave compared to female patients (RR 0.74 when female GP versus RR 0.99 when male GP).

## Discussion

### Summary

The study has four important findings: 1) when people in the workforce consult the GP with stress, it is often multifactorial and not only work-related. 2) sick leave, counselling, referral to psychologist, and medication are frequent management strategies often applied together. 3) stress is not managed equally in men and women. Compared to women, stress in men is less often considered work-related, more often managed with prescription of tranquilizers, and less often with sick leave, the latter only associated with female GPs. Finally, 4) very few cases of work-related stress are reported to the Occupational Safety- and Health- Administration or referred to a department of occupational medicine.

### Strengths and limitations

The data were collected by the involved GP assisted by EMR and were used for voluntary personal quality development incentivising proper recording. Most GPs were experienced users of the standardised questionnaire layout probably reducing recording errors [20]. The stress identification search did not include prescription records, preventing falsely increased medication frequencies.

Diagnostic coding of mental disorders has been mandatory for Danish GPs since 2014, and to improve stress identification, the GPs were given a checklist with physical, cognitive, and behavioural symptoms of stress. However, stress has no unique diagnostic code and even though several codes were used in the search, some eligible patients are likely to be missing from the study, e.g. if coded with bodily symptoms not included in the search. Overinclusion of patients seems less likely since the GPs only confirmed the stress and included the patient in around ¼ of the cases identified by the search [11]. Furthermore, the prevalence and age- and sex-distribution of patients with stress in the study matches the large labour force surveys in the UK and Denmark [1, 11, 21, 22], indicating a representative sample for countries where free GP services is associated with high frequency of contacts due to mental problems [14].

Except for the higher proportion of female GPs, the characteristics of the participants did not differ substantially from that of all GPs in the region. However, it is likely that the participating GPs were more interested in stress and may manage it somewhat differently than non-participants. Nevertheless, the associations found between stress management, patient sex, and the assessed cause of stress are likely to be generalizable.

The study included both ongoing and completed cases. Some cases unquestionably had additional management elements after the audit date. Thus, the percentage usages of the elements are underestimated. The inclusion of ongoing cases was necessary because stress cases in general practice do not have a recorded end date.

In Denmark, psychologist care is partially remunerated if the patient is referred from the GP with depression at all ages and anxiety only until the age of 38 years. This may incentivise GPs to diagnose more stressed patients with depression and explain why more patients with depression was referred to psychologist.

### Comparison with existing literature

Many studies have investigated management of common mental disorders (CMD) in general practice [14], but very few studies report specifically on stress. In this study, more than half of the patients were sick listed. The GPs had no valid information about the duration of sick leave. However, other studies indicate that these periods are generally long. In the UK on average 25.8 days are lost per episode of stress-related sick leave [1], and 54% of sick leaves lasts more than 3 weeks [6].

The GPs provided counselling to nearly half of the patients regardless of sex, age, assessed cause of stress, and coexisting mental disorders. Details on the counselling were not obtained, but the fee for counselling requires at least three and no more than seven sessions. Popular therapy forms include cognitive behavioural therapy and problem-solving therapy, both modestly effective [23]. In addition to the counselling, 39% of the patients were referred to psychologist while only 8 % had benzodiazepines. GPs in Ireland have been criticized for prescribing benzodiazepines to patients with CMD rather than using counselling [14]. An OECD report based on data from 2005 found that 30% of primary care mentally ill patients in Denmark received counselling and no medication compared to only 10% in the UK [24]. During the latest decade benzodiazepine use in Denmark has been halved (www.medstat.dk), and in this study almost half of the patients had counselling and no medication indicating a considerable shift in strategy which could maybe serve as an inspiration to other countries.

With regard to gender equality, the Global Gender Gab Report 2018 ranked Denmark number 13 and the UK number 15 out of 149 countries [25]. Nevertheless, this study indicates that men and women with stress are not perceived of and managed equally in Danish general practice. This inequality is substantial especially considering that CMD-related sick leave accounts for around 57% of all working days lost to ill health [1, 13]. Our findings of sex-differences regarding sick leave and medication points in opposite directions suggesting that they cannot be explained by sex-differences in case severity. Internationally, women have more sick leave than men even when equally ill and under similar work- and family- requirements [17]. It is generally believed that women are more tolerant of other women being on sick leave than of men. Concordantly, the found sex-difference regarding sick leave was only found if the GP was a woman. A recent Norwegian study on attitudes towards sick leave found that sick leave was more tolerated in workplaces heavily dominated by either one of the sexes [26]. Stressed women in general and in this study are often employed in the women-dominated service and health sectors while stressed men are less often employed in male-dominated workplaces [22]. So, the excess sick-leave in women might be due to sex-difference in workplaces. However, it was found only when the GP was female. Further supporting the existence of gender differences among GPs regarding attitudes towards sick-leave, a Swedish study found that in general female GPs sick-list more patients than male GPs do [27].

### Implications for research and/or practice

Even among people in the workforce, the majority of stress is not only work-related. The spectra of causes and treatment options are wide why it is pivotal that GPs are curious and enquire into multiple areas of life when choosing how to manage the individual patient. Despite the multifactorial causation of stress, sick leave is often prescribed. We need to investigate benefits and harms of this management strategy and to uncover how sick leave may best be tailored to the individual patient. Furthermore, coexisting psychiatric disorder is common which makes the clinical evaluation of the patient’s overall mental health important. Counselling may be beneficial but should be explored with regard to content and effectiveness of the actual treatment provided in general practice.

GPs should strive to perceive and manage stressed men and women equally so that choices regarding important elements of management do not depend on gender.

## Conclusions

GPs’ management of patients with stress usually involve elements of sick leave, counselling, referral to psychologist, and to a lesser extent medication. Women and men with stress are perceived of and managed differently.

## Supplementary information

**Additional file 1.** Supplementary Material. The EMR search strategy and registration form. 

## Abbreviations

CMD: Common mental disorders
CI: Confidence interval
EMR: Electronic medical records
GP: General practitioner
ICPC-2-R: International Classification of Primary Care second edition
OHSA: Occupational Safety- and Health-Administration
OECD: Organisation for Economic Co-operation and Development
RR: Relative risk
UK: United Kingdom
