# Supplementary material for: General practitioners’ management of patients with psychological stress: audit results from Denmark
Source: BMC Fam Pract. 2020 Apr 20;21:67. doi: 10.1186/s12875-020-01137-6 (PMC7168971; doi:10.1186/s12875-020-01137-6)
Supplement: Supplementary file 1 — Additional file 1. Supplementary Material. The EMR search strategy and registration form. [file 12875_2020_1137_MOESM1_ESM.docx]

**Supplementary material**

FAMP-D-19-00470

General practitioners’ management of patients with psychological stress: audit results from Denmark

Jesper Lykkegaard, Ph.D.; Anders Prior, MD, PhD.; Marianne Rosendal, MD, PhD

BMC Family Practice

**Psychological and social chapters in the International Classification of Primary Care second edition**

Titles marked with * were included in the search for patients with psychological stress.

The full ICPC classification directory can be found at: <http://www.kith.no/upload/2705/ICPC-2-English.pdf>

*Psychological chapter: Component 1 diagnoses (symptoms/complaints)*

**P01 Feeling anxious/nervous/tense***

**P02 Acute stress reaction***

**P03 Feeling depressed***

P04 Feeling/behaving irritable/angry

P05 Senility, feeling/behaving old

**P06 Sleep disturbance***

P07 Sexual desire reduced

P08 Sexual fulfilment reduced

P09 Sexual preference concern

P10 Stammering/stuttering/tic

P11 Eating problem in child

P12 Bedwetting/enuresis

P13 Encopresis/bowel training problem

P15 Chronic alcohol abuse

P16 Acute alcohol abuse

P17 Tobacco abuse

P18 Medication abuse

P19 Drug abuse

P20 Memory disturbance

P22 Child behaviour symptom/complaint

P23 Adolescent behaviour symptom/complaint

P24 Specific learning problem

P25 Phase of life problem adult

P27 Fear of mental disorder

P28 Limited function/disability (P)

**P29 Psychological symptom/complaint, other***

*Psychological chapter: Component 7 diagnoses (specific diseases/disorders)*

P70 Dementia

P71 Organic psychosis, other

P72 Schizophrenia

P73 Affective psychosis

**P74 Anxiety disorder/anxiety state***

P75 Somatization disorder

**P76 Depressive disorder***

P77 Suicide/suicide attempt

P78 Neuraesthenia/surmenage

P79 Phobia/compulsive disorder

P80 Personality disorder

P81 Hyperkinetic disorder

P82 Post-traumatic stress disorder

P85 Mental retardation

P86 Anorexia nervosa/bulimia

P98 Psychosis NOS/other

**P99 Psychological disorders, other***

*Social chapter Z: Component 1 diagnoses (symptoms/complaints)*

Z01 Poverty/financial problem

Z02 Food/water problem

Z03 Housing/neighbourhood problem

Z04 Social cultural problem

**Z05 Work problem***

Z06 Unemployment problem

Z07 Education problem

Z08 Social welfare problem

Z09 Legal problem

Z10 Health care system problem

Z11 Compliance/being ill problem

Z12 Relationship problem with partner

Z13 Partner's behaviour problem

Z14 Partner illness problem

Z15 Loss/death of partner problem

Z16 Relationship problem with child

Z18 Illness problem with child

Z19 Loss/death of child problem

Z20 Relationship problem parent/family member

Z21 Behaviour problem parent/family member

Z22 Illness problem parent/family member

Z23 Loss/death parent/fam member problem

Z24 Relationship problem friend

Z25 Assault/harmful event problem

Z27 Fear of a social problem

Z28 Limited function/disability (z)

Z29 Social problem NOS

**Symptoms that the participants were informed were associated with psychological stress:**

**English translation:**

Physical symptoms: Increased heart beat, Oppression feeling in the chest, breathlessness, dizziness, headache, muscle tensions, diffuse pain, bodily unease, nausea, stomach-ache and unease and sweating.

Cognitive symptoms: Difficulty remembering, getting easily distracted, difficulty making up ones mind, loss of perspective, difficulty solving problems and making plans, difficulty finding words, mental tiredness, reduced cognitive thinking ability and/or confusion.

Behavioural symptoms: Sleeping disturbances, nervousness, social isolation, difficulty to overcome life, reduced level of activity, severe reaction to small demands, use of psychotropic substances, restlessness, irritability, angriness and/or tendency to cry.

**Audit Project Odense registration sheet for general practice: Stress 2016.**

**
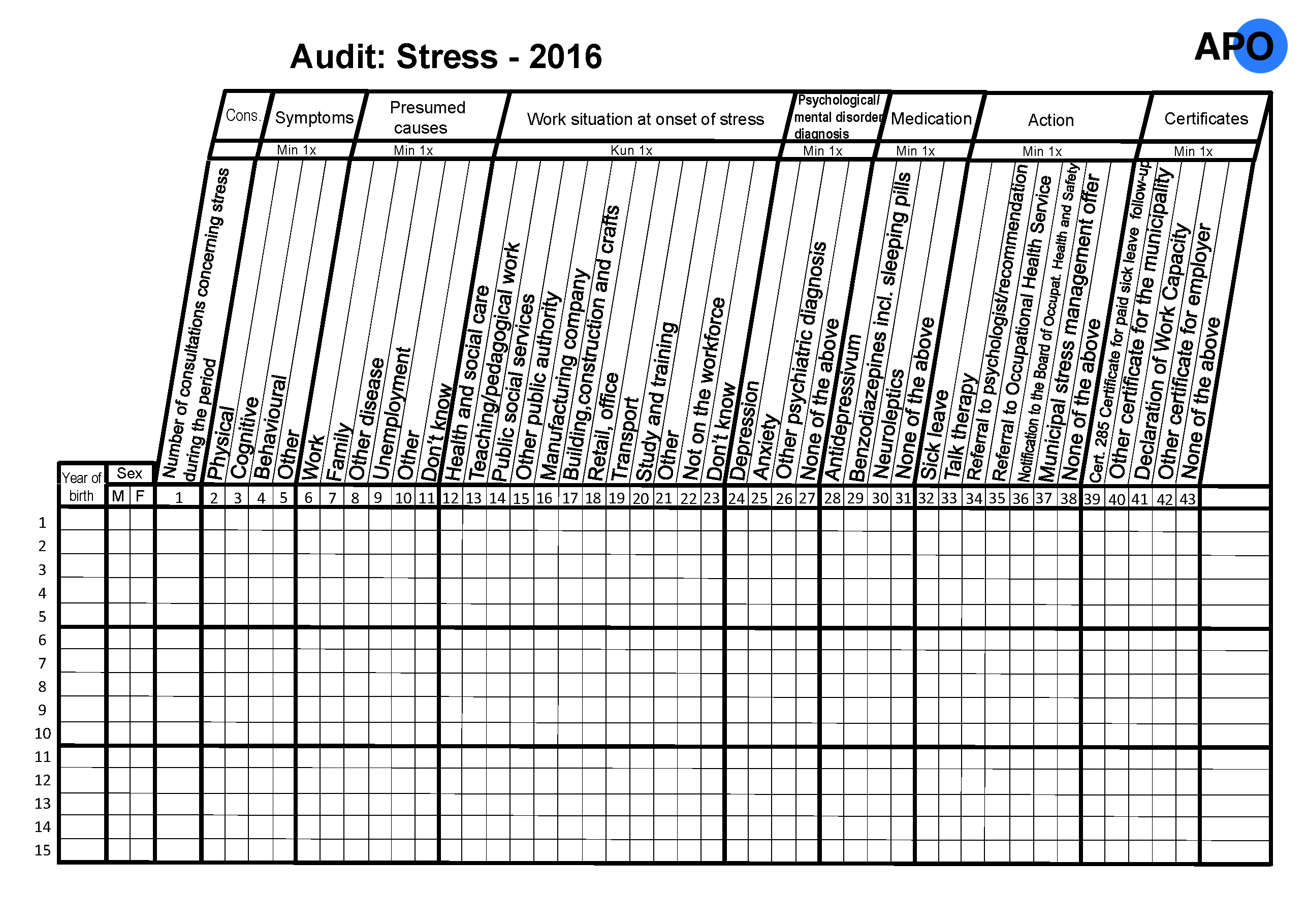
**
